# Preventive behaviours and family inequalities during the COVID-19 pandemic: a cross-sectional study in China

**DOI:** 10.1186/s40249-021-00884-7

**Published:** 2021-07-20

**Authors:** Yisheng Ye, Ruijun Wu, Yao Ge, Tao Wang, Xin Yao, Yao Yang, Chengxu Long, Fangfei Chen, Shangfeng Tang, Rui Huang

**Affiliations:** 1 School of Medicine and Health Management, Tongji Medical College, Huazhong University of Science and Technology, 13 Hangkong Road, Wuhan, China; 2 China National Center for Biotechnology Development, Beijing, China; 3 China Science and Technology Exchange Center, Beijing, China; 4 High Technology Research and Development Center, Beijing, China; 5 National Center for Science and Technology Evaluation, Beijing, China; 6 School of Pharmacy, Tongji Medical College, Huazhong University of Science and Technology, 13 Hangkong Road, Wuhan, China

**Keywords:** COVID-19, China, Preventive behaviours, Household

## Abstract

**Background:**

The coronavirus disease 2019 (COVID-19) pandemic is an international public health threat, and people's participation in disease-related preventive behaviours is the key to controlling infectious diseases. This study aimed to assess the differences in adopting preventive behaviours among populations to explore potential individual and household factors and inequalities within families.

**Methods:**

This online survey was conducted in April 2020. The directional stratified convenient sampling method was used to select 4704 participants from eight provinces in eastern, central, and western China. The questionnaire included demographic information, household variables, and five target prevention behaviours. The chi-squared test, binary multilevel model, and Mantel–Haenszel hierarchical analysis were used for data analysis in the study.

**Results:**

Approximately 71.2% of the participants had appropriate outdoor prevention, and 32.9% of the participants had indoor protection in place. Sharing behaviours (*P* < 0.001) and education level (*P* < 0.001) were positively associated with adopting preventive measures. The inhibiting effect of household crowding and stimulating effect of high household income on preventive behaviours were determined in this study. Household size was negatively associated with living area (β = -0.057, *P* < 0.05) and living style (β = -0.077, *P* < 0.05). Household income was positively associated with age (β = 0.023, *P* < 0.05), and relationship with friends (β = 0.053, *P* < 0.05). Vulnerable groups, such as older adults or women, are more likely to have inadequate preventive behaviours. Older adults (*OR* = 1.53, 95% *CI *1.09–2.15), women (*OR* = 1.37, 95% *CI *1.15–1.64), and those with more than 2 suspected symptoms (*OR* = 1.85, 95% *CI *1.07–3.19) were more likely to be affected by the inhibiting effect of household crowding, while the stimulating effect of high household income was limited in these groups.

**Conclusions:**

Inequalities in COVID-19 prevention behaviours exist between families and inadequate adoption of prevention by vulnerable groups are noteworthy. This study expands the research perspective by emphasizing the role of household factors in preventive behaviours and by focusing on family inequalities. The government should use traditional media as a platform to enhance residents’ public health knowledge. Targeted additional wage subsidies, investments in affordable housing, financial support for multigenerational households, and temporary relocation policies may deserve more attention. Communities could play a critical role in COVID-19 prevention.

**Graphical abstract:**

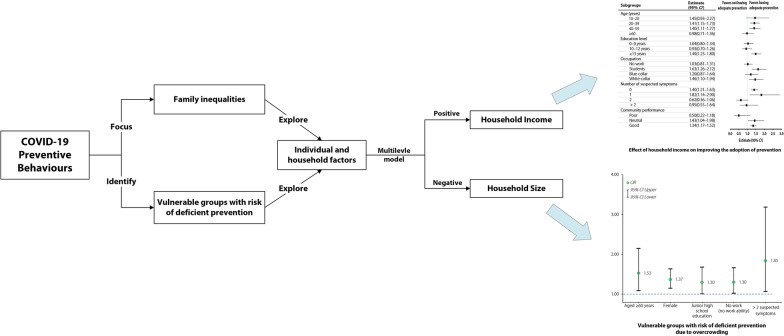

## Background

The novel coronavirus pandemic caused by the SARS-CoV-2 virus has posed serious challenges worldwide. The disease is highly contagious and has a higher potential to cause transmission of the virus before patients with COVID-19 develop symptoms [1]. As of January 26, 2021, COVID-19 has resulted in the diagnosis of more than 100 million people worldwide, with approximately 2.14 million deaths [2]. Recent studies have shown that most patients with COVID-19 still have at least one symptom at the time of discharge six months after onset, with possible long-term consequences for the patient [3]. Although more than 50 vaccines against COVID-19 have been developed and are in clinical trials, issues such as effectiveness, transport, promotion of vaccination and possible side effects have yet to be further confirmed [4]. Additionally, it takes time to produce and vaccinate tens of millions of people worldwide. China has successfully contained the outbreak by implementing multifaceted public health measures, including but not limited to urban travel restrictions, maintaining self-isolation, maintaining social distance, wearing masks outside the home, and frequent hand washing [5–7]. A study conducted in China showed that psychoneuroimmunity preventive measures in the workplace, including organizational measures such as improving workplace hygiene and company attention to physical health status, were associated with a reduction in employee psychiatric symptoms [8]. The preventive behaviours confirmed in accumulating evidence remain a critical public health measure to reduce the risk of infection and virus transmission [9, 10].

To date, research on preventive behaviours has been primarily conducted at the individual level. Existing studies include investigations of residents' disease perceptions, beliefs, and preventive behaviours [11–13], the impact of misinformation [14, 15], mental health during the COVID-19 pandemic [16, 17], and disparities in prevention practices among people of various socioeconomic statuses [18]. Aggregate transmission within the home can be a hidden danger for epidemic control. Numerous reports indicate that infected individuals in the home setting have a higher probability of transmitting the disease to susceptible populations than in most other settings [19]. Robust preventive behaviours are recommended as the primary intervention to minimize the spread of the virus in health care institutions, communities, and families [20]. A longitudinal study also suggested that promoting preventive measures can alleviate the fear and confusion of the population and protect mental health in China[21]. Therefore, understanding the adoption of preventive behaviours at the household level and exploring inequalities within families are necessary for governments in China and other countries [22, 23].

However, to our knowledge, most of the available studies on family factors have focused on COVID-19 infection and mortality [24, 25], while few studies have focused on preventive behaviours. Our study then adds slightly to previous research by focusing on how family factors, particularly household size, may affect preventive behaviours rather than infection and death and whether marginal groups within families need extra attention. Hence, this study aimed to identify differences in adopting indoor and outdoor preventive behaviours among the population and to explore the potential effects of individual factors, social support, and family settings. This research also provides policy recommendations for countries that are still trapped in the epidemic or have managed it but are at a new risk.

## Methods

### Study population and data collection

The targeted stratified convenient sampling method was employed to select residents from the eastern, central, and western regions of China. The two provinces with the highest number of patients and the province with the lowest number of patients were selected from each region based on the confirmed patients on April 1, 2020. Guangdong, Zhejiang, and Fujian provinces were selected in Eastern China. Hunan, Hubei, and Shanxi provinces were selected in the central region. Because of the similar economic and cultural conditions between Sichuan and Chongqing, only Sichuan Province was selected as the high-prevalence province in the western region. The provincial capital city and another city were selected in each province, and residents aged older than ten years in 60 households from both rural and urban areas were investigated in each city. Participants who met the following inclusion criteria participated in the survey: (1) aged 10 years or older; (2) agreed to participate in the study by accepting the online informed consent agreement. In total, 7118 residents from 1920 households in eight provinces (16 cities) participated in this survey.

The survey was conducted from April 4 to April 15, 2020. A project manager was hired in each province to coordinate and supervise the survey. After training by the project manager, six independent college investigators were hired from the same local municipalities to assist with the online survey. Each investigator was required to send electronic questionnaires via WeChat to 20 families around their local relatives, friends, or classmates. Based on the 20 households and number of family members, a unique code was generated for each questionnaire that met the survey criteria. The codes include province, city type, urban/rural type, enumerator code, household serial number, and household members. If individual enumerators could not complete the survey for 20 households, other enumerators were arranged to assist in completing the survey. Trap questions screened participants who did not answer carefully, and the quality of the questionnaire was checked by the project manager. Participants who qualified for completion were given a secret gift as a reward. The exclusion criteria were as follows: (1) an unreasonable consistency of questionnaires from the same family (e.g., the household income description was inconsistent); (2) completion of the questionnaire by the respondents in less than 7.5 min (the critical point of response time in questionnaire tests); (3) invalid questionnaire numbers (e.g., numbers with only five digits, corresponding to position values larger than the filled threshold). The final number of valid survey households was determined by coding. In total, 4704 survey participants and 1564 households were eligible for this study.

### Measurement

According to the COVID-19 clinical and community management guidelines issued by the National Health Commission of the People’s Republic of China, we designed a questionnaire to determine the residents’ preventive behaviours for COVID-19 [26, 27]. Outdoor prevention was measured using two questions: (1) whether respondents stayed home except for essential activities to prevent COVID-19 and (2) whether respondents wore masks all the time if they had to leave. The answers of "yes" and "no" were coded as "1" and "0", respectively. Outdoor prevention was measured by summing the answers' score, and a total score of 2 indicated that prevention was adopted, while a score of 0 or 1 meant the prevention was not adopted well.

In terms of indoor prevention, the measurement was made using three questions: (1) whether the respondents exercised more than before to prevent COVID-19; (2) whether the respondents focused more on maintaining personal hygiene practices (such as washing their hands frequently) since the outbreak, and (3) whether respondents tried their best to intake nutrition (including vegetables, fruits, eggs, and meat) to prevent COVID-19. The answers of "yes" and "no" were coded as "1" and "0", respectively, prevention within the home was measured by summing the answers' score, and a total score of 0, 1, or 2 indicated that indoor prevention was deficient, while 3 was adequate.

Considering the data sample and questionnaire design, the independent variables included five layers of data on demography, information, health status, social support, and family. The following were included in the study based on the research in related fields and theoretical relationship with the dependent and independent variables: number of sources of access to information regarding friends, family members, TV, newspaper, video on social media, opinions on social media, phone message, experts, and patient's experience; motivation for sharing the news of COVID-19 with family members, friends, and colleagues. The Cronbach's alpha coefficient of the questionnaire was 0.775, indicating acceptable internal consistency [28]. The details of the variables are shown in Table 1.

**Table 1:** Variables and assignments of preventive behaviours and related factors

Variables	Assignments
Dependent variables	
Outdoor prevention	0 = no; 1 = yes
Indoor prevention	0 = no; 1 = yes
Demographic factors	
Age (years)	1 = 10–20; 2 = 20–39; 3 = 40–59; 4 = ≥ 60
Sex	1 = male; 2 = female
Education level	1 = 0–9 years; 2 = 10–12 years; 3 = ≥ 13 years
Occupation	1 = no work; 2 = students; 3 = blue-collar; 4 = white-collar
Living place	1 = urban; 2 = rural
Living style	1 = living alone; 2 = living with others
Information	
Number of informative channels	1 = 0–3; 2 = 4–6; 3 = 7–9
Motivation for sharing	1 = none; 2 = low; 3 = medium; 4 = high
Health status	
Physical health status	1 = worse; 2 = same; 3 = better
Social support	
Current relationship with friends	1 = worse; 2 = same; 3 = better
Community performance	1 = poor; 2 = neutral; 3 = good
Family	
Annual household income	1 = < CNY 100 000; 2 = CNY100 000–300 000; 3 = > CNY 300 000
Household Size	1 = 1–3; 2 = 4–6; 3 = > 6

*CNY* Chinese Yuan

### Statistical analysis

Descriptive analysis, chi-squared test, binary logistic regression of the multilevel model, and Mantel–Haenszel hierarchical analysis were employed in this study. All the variables were represented as frequency distributions and percentages, and descriptive analysis was conducted on demographic characteristics and other variables. Chi-squared tests were performed to summarize the differences in outdoor and indoor prevention among those with different sociodemographic characteristics. A binary logistic multilevel regression model was employed to explore the impact of different factors on adopting outdoor and indoor prevention.

The multilevel model was employed to analyse the hierarchical data, where each low-level unit was clustered within a high-level unit. Binary logistic regression of the multilevel model was performed because the dependent variables were dichotomous. In this two-level data structure, low-level individuals were treated as level 1, representing individual-level factors such as sociodemographic information, health status, and social support, and high-level families were treated as level 2, representing household factors such as household income and household size.

An empty model with two levels without explanatory variables was first fitted using only the intercept and residuals. In the random-effects analysis, the intraclass correlation coefficient (ICC) was used to determine the intragroup correlation ICC = intercept/ (residual + intercept) in this study. If ICC = 0, the data do not have a hierarchical structure and can be reduced to a traditional one-level model. The higher is the ICC, the more prominent are the structural characteristics of the data, indicating the need for multilevel model analysis.

In fitting the two-level model, sociodemographic, information, health status, and social support factors were first added to the model in turn. Household factors were added afterward; finally, a fully adjusted model was obtained. The intercept and residuals were used as random effects in each test. Interaction term analysis was used to test the interaction between the two significant variables based on the correlations of the previous model. To further identify vulnerable groups that are more influenced by household factors, a Mantel–Haenszel hierarchical analysis of deficient prevention was conducted for each subpopulation. Statistics of the multilevel model were performed using SPSS 21.0. (IBM Corp. Armonk, NY). A *P*-value less than 0.05 was considered statistically significant.

## Results

### Characteristics of participants and adopting outdoor and indoor prevention

Approximately one-third of the participants were aged 21 to 40 years and 41 to 59 years. More than half of the participants were women, and 59.8% of the participants were married. Approximately one-fourth of the participants were students, and 29.9% of the participants could not work. Nearly half of the participants' annual household income was less than CNY 100 000; more than two-fifths of the participants had a university education, and 64% of the participants lived in the urban area; most of the participants lived with others. Approximately 71.2% of the participants performed outdoor prevention well. Regarding adopting indoor prevention, only 32.9% of the participants conscientiously performed these actions. The details are shown in Table 2.

**Table 2:** Differences in the adoption of preventive behaviours among participants with different characteristics, *n* (%)

Variables	Total	Outdoor prevention		Indoor prevention	
	No (%)	No	Yes	No	Yes
Total	4704	1354 (28.8)	3350 (71.2)	3156 (67.1)	1548 (32.9)
Age (years)					
10–20	353 (7.5)	80 (22.7)	273 (77.3)	222 (62.9)	131 (37.1)
20–39	1925 (40.9)	445 (23.1)	1480 (76.9)	1283 (66.6)	642 (33.4)
40–59	1577 (33.5)	498 (31.6)	1079 (68.4)	1044 (66.2)	533 (33.8)
≥ 60	849 (18)	331 (39.0)	518 (61.0)	607 (71.5)	242 (28.5)
* P*-value		< 0.001		0.012	
Sex					
Male	2207 (46.9)	690 (31.3)	1517 (68.7)	1490 (67.5)	717 (32.5)
Female	2497 (53.1)	664 (26.6)	1833 (73.4)	1666 (66.7)	831 (33.3)
* P*-value		< 0.001		0.564	
Education level					
0–9 years	1493 (31.7)	589 (39.5)	904 (60.5)	1105 (74.0)	388 (26.0)
10–12 years	855 (18.2)	240 (28.1)	615 (71.9)	554 (64.8)	301 (35.2)
≥ 13 years	2356 (50.1)	525 (22.3)	1831 (77.7)	1497 (63.5)	526 (36.5)
* P*-value		< 0.001		< 0.001	
Occupation					
No work	1405 (29.9)	441 (31.4)	964 (68.6)	979 (69.7)	426 (30.3)
Students	1132 (24.1)	250 (22.1)	882 (77.9)	741 (65.5)	391 (34.5)
Blue-collar	879 (18.7)	348 (39.6)	531 (60.4)	622 (70.8)	257 (29.2)
White-collar	1288 (27.4)	315 (24.5)	973 (75.5)	814 (63.2)	474 (36.8)
* P*-value		< 0.001		< 0.001	
Living place					
Urban	3009 (64.0)	713 (23.7)	2296 (76.3)	1878 (62.4)	1131 (37.6)
Rural	1695 (36.0)	641 (37.8)	1054 (62.2)	1278 (75.4)	417 (24.6)
* P*-value		< 0.001		< 0.001	
Living style					
Living alone	408 (8.7)	118 (28.9)	290 (71.1)	301 (73.8)	107 (26.2)
Living with others	4296 (91.3)	1236 (28.8)	3060 (71.2)	2855 (66.5)	1441 (33.5)
* P*-value		0.949		0.003	
Number of suspected symptoms					
0	3843 (81.7)	1136 (29.6)	2707 (70.4)	2605 (67.8)	1238 (32.2)
1	375 (8.0)	102 (27.2)	273 (72.8)	242 (64.5)	133 (35.5)
2	249 (5.3)	59 (23.7)	190 (76.3)	158 (63.5)	91 (36.5)
> 2	237 (5.0)	57 (24.1)	180 (75.9)	151 (63.7)	88 (36.3)
* P*-value		0.062		0.198	
Number of informative channels					
0–3	2015 (42.8)	579 (28.7)	1436 (71.3)	1386 (68.8)	629 (31.2)
4–6	1705 (36.2)	484 (28.4)	1221 (71.6)	1155 (67.7)	550 (32.3)
7–9	984 (20.9)	291 (29.6)	693 (70.4)	615 (62.5)	369 (37.5)
* P*-value		0.806		0.002	
Motivation for sharing					
None	134 (2.8)	84 (62.7)	50 (37.3)	101 (75.4)	33 (24.6)
Low	301 (6.4)	134 (44.5)	167 (55.5)	247 (82.1)	54 (17.9)
Middle	899 (19.1)	333 (37.0)	566 (63.0)	712 (79.2)	187 (20.8)
High	3370 (71.6)	803 (23.8)	2567 (76.2)	2096 (62.2)	1274 (37.8)
* P*-value		< 0.001		< 0.001	
Physical health situation					
Worse	463 (9.8)	137 (29.6)	326 (70.4)	305 (65.9)	158 (34.1)
Same	4048 (86.1)	1163 (28.7)	2885 (71.3)	2719 (67.2)	1329 (32.8)
Better	193 (4.1)	54 (28.0)	139 (72.0)	132 (68.4)	61 (31.6)
* P*-value		0.899		0.791	
Current relationship with friends					
Worse	294 (6.3)	76 (25.9)	218 (74.1)	205 (69.7)	89 (30.3)
Same	4082 (86.8)	1177 (28.8)	2905 (71.2)	2735 (67.0)	1347 (33.0)
Better	328 (7.0)	101 (30.8)	227 (69.2)	216 (65.9)	112 (34.1)
* P*-value		0.390		0.558	
Community performance					
Poor	170 (3.6)	65 (38.2)	105 (61.8)	133 (78.2)	37 (21.8)
Neutral	1047 (22.3)	362 (34.6)	685 (65.4)	802 (76.6)	245 (23.4)
Good	3487 (74.1)	927 (26.6)	2560 (73.4)	2221 (63.7)	1266 (36.3)
* P*-value		< 0.001		< 0.001	
Household income					
< CNY 100 000	2040 (43.4)	597 (29.3)	1443 (70.7)	1460 (71.6)	580 (28.4)
CNY 100 000–300 000	2275 (48.4)	649 (28.5)	1626 (71.5)	1477 (64.9)	798 (35.1)
> CNY 300 000	389 (8.3)	108 (27.8)	281 (72.2)	219 (56.3)	170 (43.7)
* P*-value		0.779		< 0.001	
Household size					
1–3	2169 (46.1)	604 (27.8)	1565 (72.2)	1400 (64.5)	769 (35.5)
4–6	2146 (45.6)	642 (29.9)	1504 (70.1)	1485 (69.2)	661 (30.8)
> 6	389 (8.3)	108 (27.8)	281 (72.2)	271 (69.7)	118 (30.3)
* P*-value		0.291		0.003	

*CNY* Chinese Yuan

### Differences in adopting outdoor and indoor prevention

The differences in adopting outdoor and indoor prevention among different subgroups were shown in Table 2. Age (*P*_*-outdoor*_ < 0.001; *P*_*-indoor*_ = 0.012), educational level (*P* < 0.001), occupation (*P* < 0.001), living place (*P* < 0.001), motivation for sharing (*P* < 0.001), and community performance (*P* < 0.001) were associated with adopting outdoor and indoor prevention.

Regarding the manifestations for outdoor prevention, the percentage was higher among participants were aged 10 to 20 years (77.3%), women (73.4%), those with a college education or higher (77.7%), students (77.9%), living in urban areas (76.3%), with high motivation to share (76.2%), and living in well-responsive communities (73.4%).

Concerning indoor prevention, the percentage was higher among participants were aged 10 to 20 years (37.1%), those with a college education or higher (36.5%), white-collar workers (36.8%), living in urban areas (37.6%), had 7–9 informative channels (37.5%), with high motivation to share (37.8%), living in well-responsive communities (36.3%), had household income above CNY 300 000 (43.7%), and had a household size of 1–3 people (35.5%).

### Factors associated with outdoor and indoor prevention

The estimation impact of individual- and household-level factors on the adoption of outdoor prevention was shown in Table 3. Model 1 showed the tested result of an empty model. The ICC was 0.278, and the level 2 variance of the empty model was statistically significant (*P* < 0.001); thus, 27.8% of the total variation was caused by family variation. Therefore, the data must be analysed using a multilevel modelling approach.

**Table 3:** Multilevel model of the association of household and individual settings with outdoor prevention

Variables	Β (*SE*)						
	Model 1	Model 2	Model 3	Model 4	Model 5	Model 6	Model 7
Fixed effects							
Intercept	0.716 (0.009)^‡^	0.643 (0.029)^‡^	0.702 (0.031)^‡^	0.690 (0.042)^‡^	0.695 (0.045)^‡^	0.689 (0.062)^‡^	1.067 (0.256)^‡^
Age (years, Ref: ≥ 60)							
10–20		0.091 (0.034)^†^	0.057 (0.034)	0.057 (0.034)	0.058 (0.034)	0.055 (0.034)	0.161 (0.034)^†^
20–39		0.069 (0.023)^†^	0.033 (0.024)	0.033 (0.024)	0.035 (0.024)	0.032 (0.024)	0.098 (0.024)^*^
40–59		0.043 (0.020)^*^	0.007 (0.020)	0.007 (0.020)	0.009 (0.020)	0.009 (0.020)	0.031 (0.020)
Sex (Male)		-0.036 (0.012)^†^	-0.039 (0.012)^†^	-0.039 (0.012)^†^	-0.038 (0.012)^†^	-0.037 (0.012)^†^	-0.039 (0.012)^†^
Education level (Ref: ≥ 13 years)							
0–9 years		-0.094 (0.020)^‡^	-0.076 (0.020)^‡^	-0.076 (0.020)^‡^	-0.076 (0.020)^‡^	-0.083 (0.020)^‡^	-0.336 (0.073)^‡^
10–12 years		-0.031 (0.019)	-0.030 (0.019)	-0.030 (0.019)	-0.030 (0.019)	-0.034 (0.019)	-0.168 (0.041)^‡^
Occupation (Ref: White-collar)							
No work		0.020 (0.020)	0.032 (0.020)	0.032 (0.019)	0.033 (0.019)	0.027 (0.020)	0.280 (0.089)^*^
Students		0.028 (0.021)	0.058 (0.021)^†^	0.058 (0.021)^†^	0.058 (0.021)^†^	0.052 (0.021)^*^	0.220 (0.063)^‡^
Blue-collar		-0.036 (0.022)	-0.028 (0.021)	-0.028 (0.021)	-0.028 (0.021)	-0.029 (0.021)	0.062 (0.038)
Living place (Ref: Rural)							
Urban		0.105 (0.016)^‡^	0.093 (0.016)^‡^	0.093 (0.016)^‡^	0.092 (0.016)^‡^	0.101 (0.016)^‡^	-0.071 (0.057)
Living style (Ref: Living with others)							
Living alone		-0.018 (0.023)	-0.010 (0.022)	-0.010 (0.022)	-0.011 (0.022)	-0.017 (0.023)	-0.013 (0.023)
Number of channels (Ref: 7–9)							
0–3			0.003 (0.017)	0.003 (0.017)	0.007 (0.017)	0.008 (0.017)	0.005 (0.017)
4–6			-0.002 (0.017)	-0.002 (0.017)	-0.002 (0.017)	-0.001 (0.017)	-0.004 (0.017)
Motivation for sharing (Ref: High)							
None			-0.300 (0.037)^‡^	-0.300 (0.037)^‡^	-0.287 (0.037)^‡^	-0.289 (0.037)^‡^	-0.336 (0.072)^‡^
Low			-0.159 (0.026)^‡^	-0.160 (0.026)^‡^	-0.154 (0.027)^‡^	-0.155 (0.026)^‡^	-0.201 (0.051)^‡^
Middle			-0.110 (0.017)^‡^	-0.110 (0.017)^‡^	-0.107 (0.017)^‡^	-0.107 (0.017)^‡^	-0.135 (0.029)^‡^
Physical health (Ref: Better)							
Worse				0.008 (0.035)	0.008 (0.035)	0.008 (0.035)	0.009 (0.035)
Same				0.013 (0.030)	0.013 (0.031)	0.012 (0.031)	0.012 (0.031)
Relationship with friends (Ref: Better)							
Worse					0.033 (0.033)	0.031 (0.033)	0.031 (0.033)
Same					0.005 (0.024)	0.005 (0.024)	0.006 (0.024)
Community performance (Ref: Good)							
Poor					-0.097 (0.035)^†^	-0.099 (0.035)^†^	-0.097 (0.035)^†^
Neutral					-0.058 (0.016)^‡^	-0.058 (0.016)^‡^	-0.057 (0.015)^‡^
Household income (Ref: > CNY 300,000)							
< CNY 100,000						0.053 (0.026)^*^	0.043 (0.084)
CNY100,000–300,000						0.012 (0.026)	0.014 (0.046)
Household size (Ref: > 6)							
1–3						-0.019 (0.039)	-0.175 (0.074)^*^
4–6						-0.022 (0.039)	-0.105 (0.051)^*^
Income × age							0.023 (0.011)^*^
Income × area							-0.051 (0.025)
Size × area							-0.057 (0.023)^*^
Sharing × education							0.036 (0.016)^*^
Sharing × occupation							0.023 (0.021)^†^
Random variance							
Residual	0.148^‡^	0.141^‡^	0.138^‡^	0.138^‡^	0.138^‡^	0.138^‡^	0.137^‡^
Intercept	0.057^‡^	0.054^‡^	0.052^‡^	0.052^‡^	0.051^‡^	0.051^‡^	0.050^‡^
-2 log likelihood	5501.067	5278.380	5164.743	5164.494	5144.187	5134.914	5103.785

*CNY* Chinese Yuan, *SE* standard error. ^*^ *P* < 0.05, ^†^ *P* < 0.01, ^‡^ *P* < 0.001

After adjusting for all the factors in model 6, the household and individual settings remained associated with the respondents' adoption of outdoor prevention (β = 0.689, *P* < 0.001). This result indicated that gender, education, occupation, living place, motivation for sharing, community performance, and household income were associated with outdoor prevention. Respondents who were male (β = -0.037, *P* < 0.01), had less than 9 years of education (β = -0.083, *P* < 0.001), had less motivation to share (β = -0.289, *P* < 0.001), and lived in an underprepared community (β = -0.099, *p* < 0.01) were significantly less likely to take outdoor prevention; those who were students (β = 0.052, *P* < 0.05), lived in cities (β = 0.101, *P* < 0.001), and had an annual household income less than CNY 100,000 (β = 0.053, *P* < 0.05) had a higher adoption willingness.

After comparing the relevant factors, model 7 was the optimal model and a significant negative association was found between household size and living area (β = -0.057, *P* < 0.05), implying that household size widens the gap in outdoor prevention adoption for people in different areas. Sharing motivation had a significant positive association with education (β = 0.036, *P* < 0.05) and occupation (β = 0.023, *P* < 0.01), suggesting that sharing motivation narrowed the gap in outdoor prevention among people with different education levels or occupations. There was a significant positive association between income and age (β = 0.023, *P* < 0.05).

The estimation impact of different variables on adopting indoor prevention among respondents at the individual and household levels was shown in Table 4. Model 1 shows the tested result of an empty model. The ICC was 0.252, and the level 2 variance of the empty model was statistically significant (*P* < 0.001). Twenty-five percent of the total variation was caused by family variation. Therefore, the data must be analysed using a multilevel modelling approach.

**Table 4:** Multilevel model of the association of household and individual settings with home prevention

Variables	Β (SE)						
	Model 1	Model 2	Model 3	Model 4	Model 5	Model 6	Model 7
Fixed effects							
Intercept	0.334 (0.009)^‡^	0.301 (0.030)^‡^	0.403 (0.033)^‡^	0.386 (0.012)^‡^	0.406 (0.048)^‡^	0.444 (0.064)^‡^	0.958 (0.368)^†^
Age (years, Ref: ≥ 60)							
10–20		0.065 (0.036)	0.065 (0.036)	0.027 (0.036)	0.026 (0.036)	0.026 (0.036)	-0.138 (0.021)^*^
20–39		-0.002 (0.025)	-0.002 (0.025)	-0.035 (0.025)	-0.031 (0.025)	-0.029 (0.025)	-0.138 (0.017)^†^
40–59		0.016 (0.021)	0.016 (0.021)	-0.015 (0.021)	-0.011 (0.021)	-0.013 (0.021)	-0.060 (0.017)^*^
Sex (Male)		-0.007 (0.013)	-0.007 (0.013)	-0.013 (0.013)	-0.012 (0.013)	-0.012 (0.013)	-0.011 (0.013)
Education level (Ref: ≥ 13 years)							
0–9 years		-0.088 (0.022)^‡^	-0.088 (0.022)^‡^	-0.073 (0.022)^†^	-0.071 (0.022)^†^	-0.062 (0.022)^†^	0.009 (0.041)
10–12 years		-0.019 (0.021)	-0.019 (0.021)	-0.018 (0.021)	-0.019 (0.021)	-0.013 (0.021)	0.031 (0.029)
Occupation (Ref: White-collar)							
No work		-0.002 (0.021)	-0.002 (0.021)	0.012 (0.021)	0.014 (0.021)	0.018 (0.021)	0.137 (0.058)^*^
Students		-0.009 (0.023)	-0.009 (0.023)	0.030 (0.023)	0.030 (0.023)	0.033 (0.023)	0.128 (0.048)^†^
Blue-collar		0.015 (0.023)	0.015 (0.023)	0.022 (0.023)	0.019 (0.023)	0.018 (0.023)	0.078 (0.036)^*^
Living place (Ref: Rural)							
Urban		0.098 (0.017)^‡^	0.098 (0.017)^‡^	0.088 (0.016)^‡^	0.086 (0.016)^‡^	0.077 (0.017)^‡^	0.077 (0.017)^‡^
Living style (Ref: Living with others)							
Living alone		-0.069 (0.024)^†^	-0.069 (0.024)^†^	-0.063 (0.024)^†^	-0.066 (0.024)^†^	-0.063 (0.024)^†^	-0.185 (0.063)^†^
Number of channels (Ref: 7–9)							
0–3			-0.066 (0.018)^‡^	-0.066 (0.018)^‡^	-0.059 (0.018)^†^	-0.059 (0.018)^†^	-0.272 (0.018)^†^
4–6			-0.049 (0.018)^†^	-0.049 (0.018)^†^	-0.048 (0.018)^†^	-0.049 (0.018)^†^	-0.156 (0.018)^†^
Motivation for sharing (Ref: High)							
None			-0.103 (0.040)^†^	-0.104 (0.040)^†^	-0.088 (0.040)^*^	-0.087 (0.040)^*^	-0.099 (0.040)^*^
Low			-0.166 (0.028)^‡^	-0.166 (0.028)^‡^	-0.160 (0.028)^‡^	-0.162 (0.028)^‡^	-0.161 (0.028)^‡^
Middle			-0.145 (0.018)^‡^	-0.145 (0.018)^‡^	-0.142 (0.018)^‡^	-0.141 (0.018)^‡^	-0.143 (0.018)^‡^
Physical health (Ref: Better)							
Worse				0.038 (0.037)	0.049 (0.038)	0.052 (0.038)	0.054 (0.038)
Same				0.016 (0.032)	0.017 (0.033)	0.020 (0.033)	0.020 (0.033)
Relationship with friends (Ref: Better)							
Worse					-0.017 (0.036)	-0.017 (0.036)	0.160 (0.095)
Same					-0.006 (0.026)	-0.005 (0.026)	0.085 (0.052)
Community performance (Ref: Good)							
Poor					-0.085 (0.037)^*^	-0.088 (0.037)^*^	-0.214 (0.066)^†^
Neutral					-0.091 (0.016)^‡^	-0.091 (0.016)^‡^	-0.158 (0.032)^‡^
Household income (Ref: > CNY 300,000)							
< CNY 100,000						-0.077 (0.028)^*^	-0.032 (0.124)
CNY100,000–300,000						-0.059 (0.027)^†^	-0.033 (0.068)
Household size (Ref: > 6)							
1–3						0.037 (0.038)	-0.256 (0.145)
4–6						0.002 (0.039)	-0.144 (0.080)
Income × friends							0.053 (0.027)^*^
Size × living style							-0.077 (0.037)^*^
Education × occupation							0.017 (0.008)^*^
Community × channels							-0.039 (0.016)^*^
Random variance							
Residual	0.166^‡^	0.164^‡^	0.161^‡^	0.161^‡^	0.162^‡^	0.162^‡^	0.162^‡^
Intercept	0.056^‡^	0.052^‡^	0.049^‡^	0.049^‡^	0.046^‡^	0.045^‡^	0.045^‡^
-2 log likelihood	5939.964	5842.756	5739.686	5738.392	5705.509	5693.223	5665.025

*CNY* Chinese Yuan, *SE* standard error. ^*^ *P* < 0.05, ^†^ *P* < 0.01, ^‡^ *P* < 0.001

After adjusting for all factors in model 6, household and individual settings remained associated with adopting home prevention for the respondents (β = 0.444, *P* < 0.001). This result indicated that education level, living place, living style, number of informative channels, sharing motivation, community performance, and household income were associated with indoor prevention. Respondents who had junior high school education and lower (β = -0.062, *P* < 0.01), lived alone (β = -0.063, *P* < 0.01), had fewer informative channels (β = -0.059, *P* < 0.01), had less motivation to share (β = -0.087, *P* < 0.05), lived in an underprepared community (β = -0.088, *P* < 0.05), and had a household income less than CNY 300 000 (β = -0.077, *P* < 0.05) had a significantly lower adoption willingness for indoor protection. Additionally, the respondents who lived in urban areas (β = 0.077, *P* < 0.001) showed better adoption.

After comparing the relevant factors, model 7 was the optimal model. A negative association was found between household size and living style (β = -0.077, *P* < 0.05), widening the adoption gap for people with different living styles. Significant positive associations were found between income and friends (β = 0.053, *P* < 0.05). Thus, income has a significant moderating effect on the completion of indoor protection for different friend relationship populations, reducing the gap in adoption. In addition, education also has such an effect on different occupational populations (β = 0.017, *P* < 0.05). Community performance was negatively associated with informative channels (β = -0.039, *P* < 0.05).

Thus, a higher education, a higher motivation to share, and higher household income have a facilitative effect on preventive behaviours, while overcrowded households have an inhibiting effect.

### Groups relatively vulnerable to overcrowded and intergenerational cohabitation

**Fig. 1:**
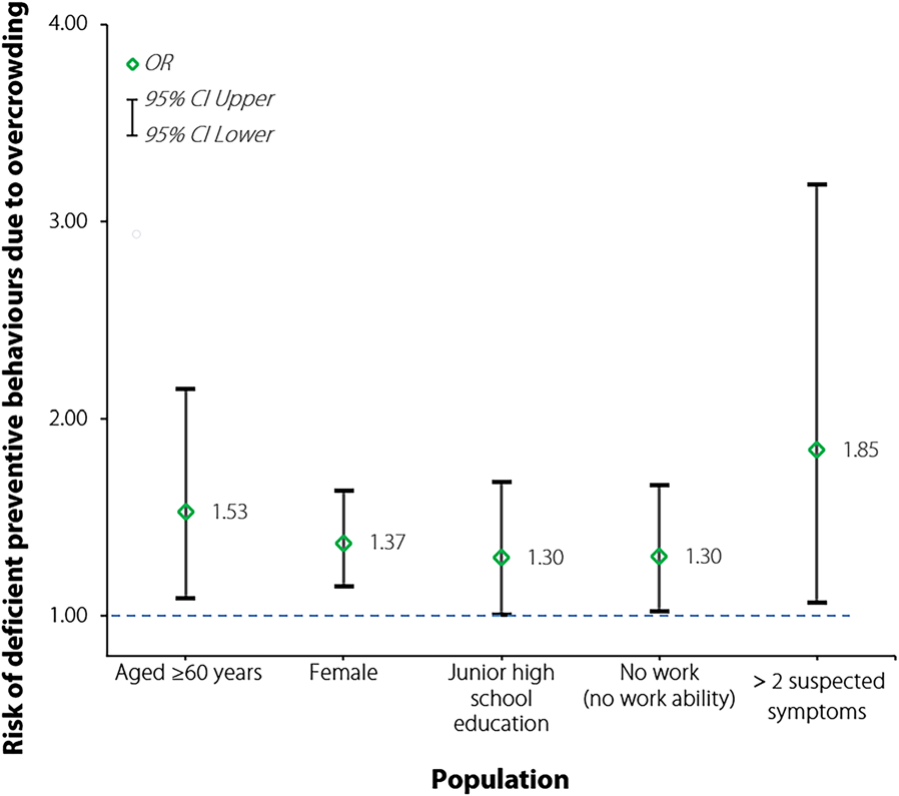
Vulnerable populations and a higher risk of deficient prevention. *OR* odd ratio, *CI* confidential interval

A larger household size implied overcrowding and intergenerational cohabitation. The study also identified vulnerable populations including the respondents who were aged older than 60 years (*OR* = 1.53, 95% *CI *1.09–2.15), female (*OR* = 1.37, 95% *CI *1.15–1.64), no work or no work ability (*OR* = 1.30, 95% *CI *1.02–1.66), a had junior high school education (*OR* = 1.30, 95% *CI *1.01–1.68), and had more than 2 suspected symptoms (*OR* = 1.85, 95% *CI *1.07–3.19). These individuals were more vulnerable to the adverse effects of considerable household size on preventive behaviours, leading to inadequate prevention. The details are shown in Fig. 1.

**Fig. 2:**
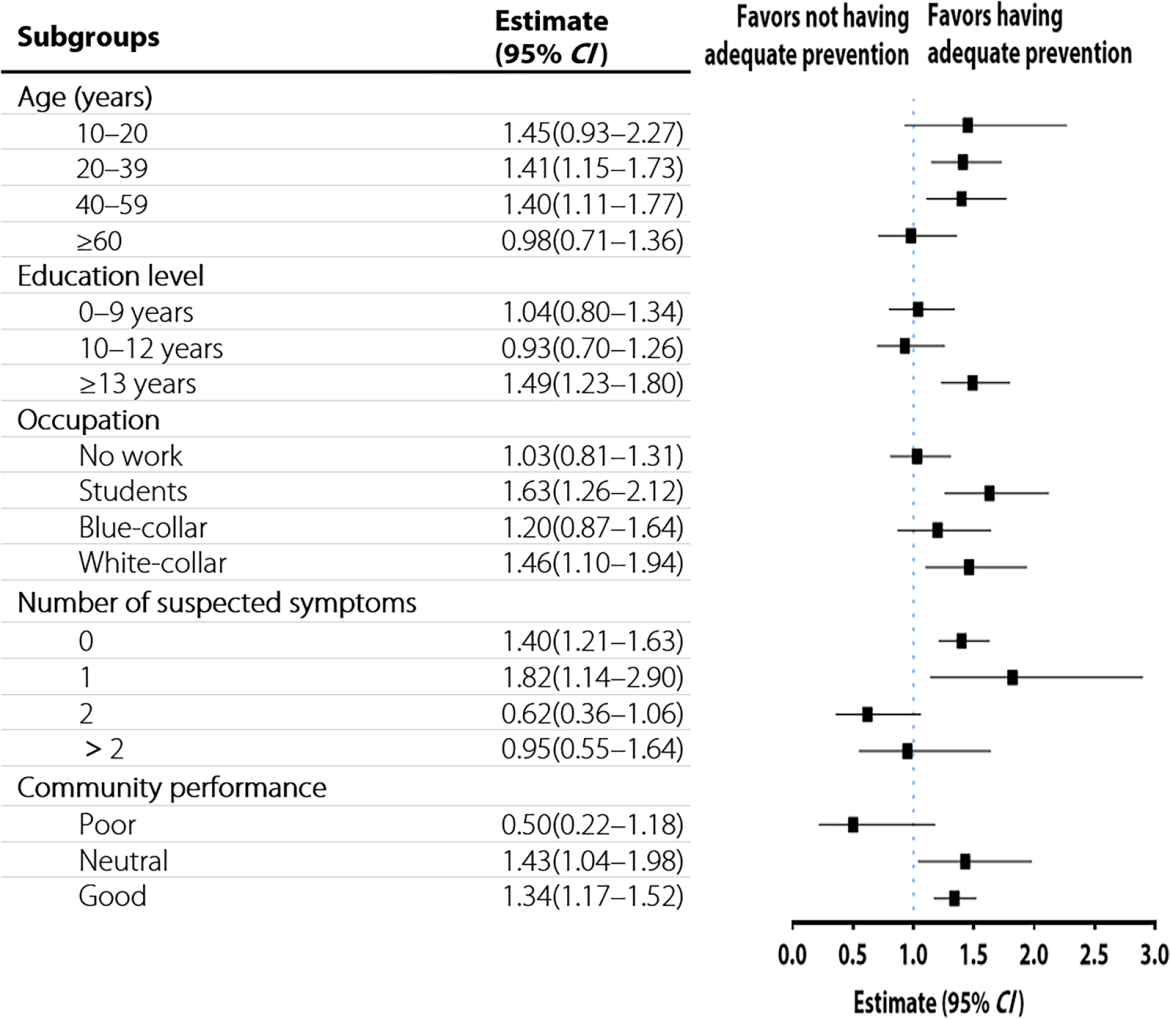
Effect of household income on improving the adoption of prevention. *CI* confidential interval

The probability of having adequate prevention for participants with a household income greater than CNY 100,000 (ref: a household income less than CNY 100,000) was illustrated in Fig. 2. Although an increased household income played an effective role in promoting preventive behaviour, it had a relatively limited impact on vulnerable groups, such as residents aged ≥ 60 years (*OR* = 0.98, 95% *CI *0.71–1.36), those without work (*OR* = 1.03, 95% *CI *0.81–1.31), those with two suspected symptoms (*OR* = 0.62, 95% *CI *0.36–1.06), and those living in communities with poor coping and handling capacities during the epidemic (*OR* = 0.50, 95% *CI *0.22–1.18). These individuals are less likely to perceive the stimulating effect of high household income on preventive behaviours; instead, they are at a high risk of being marginalized, leading to inadequate preventive behaviours.

## Discussion

### Underutilization of preventive behaviours

The present study found that the utilization of indoor protection (32.9%) was lower than that of outdoor protection (72.1%), results similar to those of a previous study [29]. Indoor preventive behaviours could easily be overlooked for various reasons. Exercise and nutritional supply were more focused on immunity improvement, but its effect was not as immediate [29]. Weak perception by the residents was manifested by a lack of identification with the information, leading to superficial and ineffective information processing [13, 30]. The population's views on preventive behaviours may influence their willingness to adopt preventive measures. For cultural reasons, Poles hold ambivalent views concerning face masks and generally find it challenging to accept the need to use them. The Chinese are concerned with social coherence and collective order [31]. Future studies might consider the perception of the COVID-19 vaccine in different populations, the willingness to be vaccinated and influencing factors such as perceived susceptibility [32]. Dilemmas such as shortages of clean water and food supplies during an epidemic could make it difficult for authorities to maintain the nutritional supply and good hygiene practices of the population [33, 34].

Therefore, expanding the application of hand sanitizing by strategically placing hand sanitizers in high-traffic public places (e.g., malls, restaurants, and libraries), promoting the dissemination of up-to-date information on regulations and policies for COVID-19 prevention through the mass media [35], providing training programs for medical students that includes both theoretical and contextual approaches [36], and targeted communication to the public to improve compliance with hygiene behaviours could be worth the government’s attention [37]. Public health workers must increase public identification of preventive behaviours to foster daily exercise and hand-washing habits in the wake of the COVID-19 pandemic.

### Education and sharing motivation

The higher is the level of education, the greater is the motivation to share news and the higher is the utilization of preventive behaviour. This finding was consistent with that noting that adult education was associated with health behaviours [38, 39]. Related studies have demonstrated that college-educated individuals have better health habits and higher awareness of self-protection. This finding might be explained by the excellent ability to retrieve and understand health information and the strength of convictions in controlling the disease [40]. In a survey of Chicago, those with low health literacy were less likely to believe they could be infected and were less willing to adopt corresponding preventive behaviours [41]. This finding demonstrated the helplessness of these individuals to change their social environment and their lack of clear and actionable public health communication [40, 42].

Sharing behaviours promoted the adoption of preventive behaviours among residents. The more concerned residents are regarding the outbreak, the more likely they will share information with others [43]. This finding might be related to their perceived effectiveness of preventive behaviours [44]. Additionally, as a social activity, sharing behaviour enhances social support to residents and alleviates potential mental health problems [45]. Thus, targeted education for populations with low health literacy may be a worthwhile endeavour. Regularly updated public health actions and sufficient practical information on how to respond could be worth the government’s attention. Easy-to-understand and straightforward language could enhance the population's translation and utilization of public health knowledge. The new social media is better suited as a new platform for people to support each other and share problems and solutions during isolation.

### Household factors and inequalities

As expected, preventive behaviours differed significantly across households. Individuals living in larger households were less likely to adopt appropriate precautions, consistent with previous findings [46]. People living in densely populated communities were less likely to have the space and financial capacity to practice social alienation and self-isolation [33]. Overcrowded living conditions without sanitation facilities during the pandemic also made maintaining hand hygiene nearly impossible [47]. This finding could be associated with overcrowding in households and cohabitation of different generations. Overcrowding in accommodations could cause several problems, such as an inadequate food supply, an unbalanced diet, a lack of exercise, and low cognitive stimulation [18]. Additionally, it significantly reduced the well-being of residents' lives [48], leading to a cascade of physical and mental health problems, which lower the level of prevention in the population.

The results also showed that higher household income could promote the adoption of preventive behaviours among residents. Our finding was in line with the existing studies that higher socioeconomic status groups were more likely to adopt appropriate preventive measures [49]. This finding might be related to higher-income groups being less prone to financial hardship due to epidemics [50]. They could focus on the quality of life as much as possible and be more likely to develop a good sense of protection. By contrast, low-income people were six times less likely to be able to work from home and three times less likely to be able to self-segregate [12]. Possible reasons for this observation were that low-income people tend to be employed in occupations that do not offer work-at-home opportunities (e.g., nursing services, transportation, food, and restaurants) [18].

Interestingly, the effects of overcrowding on preventive behaviours were not equal across individuals. Specifically, among our participants, some vulnerable groups were more susceptible to the negative effects of intergenerational cohabitation on preventive behaviours, including older participants, women, and the frail and sick. Worse still, although increasing household income was a favourable promoter of preventive behaviour, the effect may have been limited among these vulnerable groups.

Reasons for the inequality may be multifaceted. First, Chinese culture is one in which older adults are accustomed to "leaving good things to the next generation rather than themselves", and intergenerational conflicts require them to focus on their children at the expense of themselves [51]. Second, this inequality was also exacerbated by the development and implementation of "ageist" policies that prioritize resources based solely on the age of the patient [52]. In the South African pandemic, women were more likely to lose their jobs, take on additional childcare responsibilities, and face gender-based wage gaps [53]. Health risks coexist with socioeconomic vulnerability, indicating that a weak health status may exacerbate existing financial instability and make people more vulnerable to the negative effects of COVID-19 [54]. These groups are marginalized in society and families and are largely excluded from the resources needed for social protection and to minimize infection with the virus. The third point was that the Internet could efficiently deliver information in an epidemic. However, because of high costs, digital literacy and technical support are low. Many socioeconomically disadvantaged groups had multiple barriers to accessing emerging digital technologies, and they may find it difficult to consider their own needs [55]. Fourth, discrimination and stigma against the elderly, poor, and lower classes also increased during the pandemic; thus, vulnerable groups may face increased psychological stress [52]. Frail older adults mostly accounted for the increased household size while not perceiving the benefits of higher household income. Future research may consolidate the specific relationships between household factors, preventive behaviours, morbidity, and mortality among vulnerable groups.

Hence, providing additional wage compensation to the poor, such as a one-time subsidy of 2 months of the minimum living wage, could be worth the government’s attention. Suitable, affordable housing could be considered a long-term investment that provides financial support for multigenerational families and facilitates temporary relocation [56]. A top-down, one-size-fits-all approach derails countless well-meaning solutions, and there is a greater need to address real needs through local governance models. Intersectoral collaboration at the grassroots level in pandemic prevention may be worthwhile to consider. Additionally, community service workers perform well in intersectoral collaboration [57]. Communities are the key to establishing regional networks and providing precise assistance. Local trusted communication channels (e.g., reputable community leaders and teachers) and volunteers can be used to deliver information, run errands, procure and deliver food, and provide timely medicine [58]. Volunteers can visit multiple families on the same day to collect and give feedback on the real family situation. Existing resources can be mobilized to provide health workers and volunteers in rural areas with the knowledge, skills, and materials to provide lean management for at-risk communities [59]. The needs of marginalized groups can be addressed and resources allocated fairly and effectively to the appropriate people. Regular phone or Internet meetings with family members may be helpful for individuals to enhance intergenerational communication and maintain a healthy mental state.

Our study expands the research perspective by emphasizing the role of household factors in preventive behaviour and by focusing on the inequalities that exist. It also emphasizes that civil society must hold the state responsible for distributing social protection where it is most needed during and after COVID-19. However, this study still has some limitations. First, this study is a cross-sectional survey and does not reflect the causal relationship between the data. Second, this study used electronic questionnaires for data collection, and those who did not have access to the Internet were not adequately surveyed. Third, the respondents may have some subjective bias when answering specific questions such as those concerning wearing masks and washing hands. Fourth, variables such as preventive measures and social support are not sufficiently comprehensive and could be further improved and supplemented.

## Conclusions

Adopting indoor protection was insufficient in China compared with adopting outdoor protection. Age, education level, occupation, place of residence, motivation to share, and community performance were associated with adopting preventive behaviours. Educational attainment and motivation to share were positively associated with adopting preventive behaviours. Among the household factors, household income played a facilitating role, while a larger household size limited the adoption of preventive behaviours to some extent. Older adults, women, unemployed individuals, and those with underlying diseases were more vulnerable to the negative effects of intergenerational cohabitation, while the facilitating effect of higher household income was fairly limited among these vulnerable groups. Enhancing public education to improve residents' conversion and recognition of public health knowledge and providing additional financial subsidies and housing policies to help vulnerable groups deserve the attention of authorities. Communities can play a greater role in COVID-19 prevention and response.

## Data Availability

The datasets used and/or analysed during the current study are available from the corresponding author on reasonable request.

## Abbreviations

CNY: Chinese Yuan
*SE*: Standard error
*OR*: Odd ratio
*CI*: Confidential interval
